# Monthly Summary

**Published:** 1876-08

**Authors:** 


### MONTHLY SUMMARY.

---

*The Coca or Cuca Leaf and its Effects.*—The alleged use of this plant by Mr. Weston, in his great pedestrian feats has brought the subject before the medical profession of England, and Mr. Robert Christison and Mr. G. F. Dowdeswell have furnished some interesting facts on the properties and action of the leaf. The early history of Peru, says Mr. Christison, informs us that Cuca was used in Peru at the time of its conquest by the Spaniards. Poppig, a German naturalist who lived in Peru for five years, after observing the habit of chewing the Cuca leaves by the inhabitants, came to the conclusion that its use is as seductive and injurious to the health, mind, and morals, as drinking or opium eating. Its victims lie in open fields dreaming beautiful dreams, remaining out all night, indifferent to cold or rains. In the end, however, the stomach gives way, the face becomes haggard, and the victim dies of all sorts of maladies. Other authorities think that the accounts given by Poppig are much exaggerated. Dr. von Tschudi, in his visit to Peru, found it relieved the difficult breathing which is felt in ascending eminences. He thought that the moderate use of Cuca might be conducive to health. Without it, the poorly-fed Peruvian Indians would be incapaciated from performing their labor. The plant from which the leaves are obtained thrives best in the elevated forests of the Andes. In time it is covered with delicate white flowers, which are succeeded by red berries. The leaves can be stripped from the plant three times a year, and are at once thoroughly dried. When the packages of Cuca are opened, they emit a powerful tea-like odor. Mr. Christison has tried the effects of the leaves upon himself. He has taken long and fatiguing walks, living at the same time after his usual manner; then he has repeated the walks for even greater distances, and when overcome with fatigue, at some resting place, he has chewed thoroughly, and swallowed eighty grains of Cuca. No real effects were observed until he went out of doors and resumed his rapid walking; then all sense of weariness disappeared, and he could walk, not only with ease but elasticity. At the end of the walk the pulse was 90 ; and in two hours fell to 72. At dinner-time there was neither feeling of hunger nor thirst, after abstaining from food for nine hours ; but upon dinner appearing, ample justice was done to the meal. No unpleasant effects were felt the next day. Mr. Dowdeswel in an article in the *Lancet* for April 29th, 1876, has the same idea as to the great and wonderful powers of endurance gained by its use.—*British Medical Journal.*

---

*Boracic Acid in the Treatment of Ringworm.*—Surgeon Mayor Watson (*Indian Med. Gazette*) has lately used boracic acid with great success as an external application in the treatment of vegetable parasitic diseases of the skin. In the different forms of tinea (T. tonsurans and circinata,) and in that very troublesome form of the disease which effects the scrotum and inner side of the upper part of the thighs of many Europeans in India, its application acts like a charm. A solution of a drachm of the acid to an ounce of water, or as much as the water at ordinary temperatures, will take up, is employed. The affected parts should be well bathed in the solution twice daily, some little friction being used, and the solution allowed to dry on the part.—*Am. Jour. Med. Science.*

---

*How do Nerves Transmit Impulses.*—Dr. McDonnell, (*Medical Times,*) answers this question as follows : "I conceive that the various peripheral expansions, of sensitive nerves take up undulations or vibrations and convert them into waves capable of being propagated along nervous tissue. Thus the same nerve tubule may be able to transmit along it vibrations differing in character, and hence giving rise to different sensations; and consequently the same nerve tubule may in its normal condition transmit the wave which produces the idea of simple contact or that which produces the idea of heat, or again the same nerve tubules in the optic nerve which propagate the undulation of red may also propagate in normal vision those which excite the idea of yellow or blue, and so for the other senses.

---

*How to Check Coughs.*—Dr. Brown Sequard, in his late Boston lectures, says that there are many facts which show that morbid phenomena of respiration can always be stopped by the influence of arrest. Coughing. for instance, can be stopped by pressing on the nerves of the lip in the neighborhood of the nose. A pressure there may prevent a cough when it is beginning. Sneezing may be stopped by the same mechanism. Pressing in the neighborhood of the ear, right in front of the ear, may stop coughing, It is also preventive of hiccough, but much less so than of sneezing or coughing. Pressing very hard on the top of the mouth inside is also a means of stopping coughing. And, he adds, that the will has immense power there. There was a French nurse who used to say, "The first patient who coughs here will be deprived of his food to-day." It was exceedingly rare that a patient coughed then.—*Baltimore Physician and Surgeon.*

---

*Boracic Acid.*—Briefly to sum up the advantages of Boracic Acid :—

1. It is an antiseptic which does not irritate and inflame, and so allows the natural processes of healing to go on without much interruption.

2. It is exceedingly simple in its application, and can be used apart from all the details required by a thoroughly antiseptic method.

3. It can be used in the shape of the lint lotion, cotton wool, etc., in combination with most other methods of treatment.

4. Its cost is trifling ; and though this is of secondary importance, it is a feature of the treatment which will recommend its employment in workhouse, infirmaries and in dispensary and parish practice.—*Med and Surg. Reporter.*

---

*Dressing for Burns.*—Dr. Q. C. Smith, extols, in the *Pacific Medical and Surgical Journal,* the following dressing as the best for burns :—

Mix subnit. bismuth with pure honey until it forms a thick paste, spread the mixture plentifully over the burned surface and the parts near adjoining, as soon as possible after the burn occurs. Then cover the parts thickly with cotton-wool batting and bind closely. In the majority of cases, this dressing should not be removed for three or five days, when the parts should be immersed in water until the dressing is very soft and easily removed. The same dressing should be immediately renewed. —*Med. and Surg. Reporter.*

---

*Toothache.*—I have for a long time very frequently been successful in giving patients relief by stopping the hollow tooth by a paste made in the palm of the hand, by dropping on to a good pinch of the bi-carbonate of soda as much tincture of opium or of the vinum opii as the soda will take up, working the whole into a paste, and putting into the tooth.—Dr. W. B. Holderness, *Practitioner.*

---

*Proportion of Medical Men to Population in England.*—In the English army there is one surgeon to every 202 men; in the general population only one medical practitioner to every 1,276 men, women and children. At the census of 1871 the number of physicians and surgeons was 14,684, under-graduates and assistants 3,116. In this country there is on the average about one physician to 500 patients, and yet we are told that there is abundance of room for the annual crop of graduates.—*Med. Record.*

---

*Anœsthesia of Drunkenness Recommended.*—Professor Lynk, of Terre Haute, Indiana, announces in the Cincinnati *Lancet and Observer*, the wonderful discovery that the best of all anæsthetics is alcohol, drank to the extent of producing coma. Several cases are described in illustration. One was that of a lady who had a "tumor on her side." She was prepared for its removal by taking two ounces of brandy every ½ hour till four doses had been imbibed. Half an hour after the last dose he found her sleeping and quite unconscious, and proceeded to remove the tumor. The operation lasted fifteen minutes; during which she moaned and talked unintelligibly. She vomited once, as she had done once or twice before his arrival. The next day she was sitting up, feeling quite well. He is so delighted with his discovery that he predicts the universal adoption of it within ten years.—*Pacific Med. & Surg. Journal.*

---

*Female Doctors in the National Association.*—In a sketch of the proceedings of the American Medical Association, contained in the *N. Y. Med. Record*, we find the following in connection with the calling of the roll:

"On the reading of the name of Sarah Hackett Stevens, representing the Illinois State Society, Dr. Brodie, of Detroit, moved that and all such names be referred to the Judicial Council. A motion that this resolution be laid upon the table was carried by a large vote, amid considerable applause."

"The President asked if the vote was intended to recognize her right to a seat, when loud cries of 'Yes,' and cheers, emphatically answered the question."

This is the quietest and greatest victory ever gained by the sex.—*Pacific Med. and Surg. Journal.*

---

*Relation of Alcohol to Animal Heat and Muscular Power.—By Dr. Richardson.*—From which the following are logical deductions: Alcohol is disposed of in the living body by other processes than simple direct elimination. But alcohol is not a builder of any tissue, even a fatty tissue. During every stage of its administration, except the first, alcohol reduces animal temperature. Thus it is not burned after the manner of food that supports combustion. It is decomposed into secondary products by oxidation, at the expense of the oxygen, which ought to be applied to the natural heating of the body. Further as animal heat is reduced, so is the excitability of muscle, until at last, as narcotism is developed by the spirit, the muscular power is brought down to the extreme of prostation.—*Pacific Med. and Surg. Journal.*

---

*Neuralgia Relieved by Morphia and Atropia Combined.*—Dr. J. S. Jewell strongly recommends for the relief of this malady the use of a combination of morphia and atropia. The proportion is one grain of the latter to sixteen to twenty-four of the former, to an ounce of distilled water. This is given hypodermically in doses suitable to relieve pain. He says that he knows of no combination of narcotics so valuable in cases of neuralgia, and at the same time attended by so few ill consequences.—*Detroit Review of Med and Phar.*

---

*The Skin and Bodily Temperature.*—The following conclusions are given by Dr. Winternitz on the importance of the cutaneous function with regard to the heat of the body. His paper is given in Stricher's *Jahrbucher.*

1. The increase and diminution of loss of heat from the skin can be stated approximately in figures.
2. Experiments show that the giving off of heat may vary more than 60° downward, and more than 92° upward.
3. Such a variation in loss of heat can compensate variations in the production of heat three times the normal amount.
4. The discoverable variations in the loss of heat suffice to explain the constancy of temperature, so far as it exists under the usual cooling and warming influences.
5. The diminution in the loss of heat, consequently its retention, after previous loss, even if the production remain constant, is sufficient to replace in a short time the loss of temperature.
6. A diminution in the loss of heat alone can in many cases explain a febrille rise of temperature.
7. The possible increased loss of heat of more than 92° explains the frequently observed rapid disappearance of fever.
8. There is, consequently, no doubt that one of the most important factors in the regulation of temperature is to be found in the functions of the skin.—*Med. and Surg. Reporter.*

*Dangers of Breathing by the Mouth.*—Dr. Guye [*Archiv fur Ohrenheilkunde—London Med. Record,* March 15, 1876,] directs attention to the evils of breathing by the mouth. To appreciate these it must be remembered that the functions of the nose in respiration are three fold.

1. The olfactory sense secures it against the entrance of impure air.

2. The moisture of the nasal passages gives a certain degree of aqueous saturation to the inspired air, the contact of which is thus rendered less irritating to the mucous membrane of the throat and larynx.

3 The inequalities of the organ retain solid particles suspended in the air, which is proved by the quantity of dust sometimes found accumulated in the nostrils. These functions are all lost in breathing by the mouth. Further, the contact of dry air soon produces circulatory troubles in the pharyngeal region, and even an habitual catarrh, susceptible of easy transmission by continuity to the eustachian tube and cavity of the tympanum. Granular adenoid pharyngitis often has this origin. Niemeyer believed that attacks of pseudo-croup in children have their origin in dryness of the glottis produced by oral respiration. To enable the patient to breathe through the nose we must restore the nose to its proper condition, and then in children stop the mouth by an impervious respirator. Many cases of catarrhal deafness were cured in this way alone.—*Jour. Materia Medica.*

---

*Medical Diploma for Women.*—The *British Medical Journal* states that the council of the Royal College of Surgeons of England has arrived at the important decision to admit women to examination for its license in midwifery. This diploma will entitle them to a place on the *Medical Register,* and will give them a legally recognized position in this country as practitioners in the obstetric department of medicine and surgery. The clause in the College Charter under which the right to admission has been claimed, was, it appears, expressly framed by the use of the word "persons," to meet the case of female as well as of male practitioners; and the college has been advised that it could not legally refuse to admit duly educated women to examination for this diploma.

The St. Louis Eclectic Medical College opens its doors and offers every encouragement to women of noble purposes and steady aims.—*Ed.—Journal Materia Medica.*

---

*A New Dental Hospital* is about to be established in Dublin, after the plan of the London Dental Hospital.

